# Experimental Determination of a Single Atom Ground State Orbital through Hyperfine Anisotropy

**DOI:** 10.1021/acs.nanolett.2c02783

**Published:** 2022-10-28

**Authors:** Laëtitia Farinacci, Lukas M. Veldman, Philip Willke, Sander Otte

**Affiliations:** † Department of Quantum Nanoscience, Kavli Institute of Nanoscience, Delft University of Technology, 2628 CJDelft, The Netherlands; ‡ Physikalisches Institut, Karlsruhe Institute of Technology, 76131Karlsruhe, Germany

**Keywords:** scanning tunneling microscopy, electron spin resonance, hyperfine interaction, vector magnetic field, single-atom magnetism, magnetic sensing

## Abstract

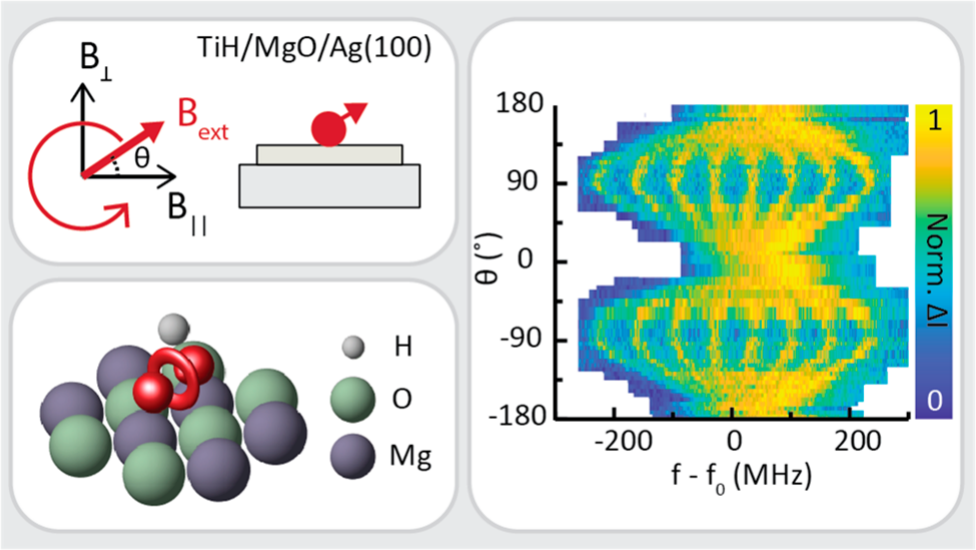

Historically, electron spin resonance (ESR) has provided excellent insight into the electronic, magnetic, and chemical structure of samples hosting spin centers. In particular, the hyperfine interaction between the electron and the nuclear spins yields valuable structural information about these centers. In recent years, the combination of ESR and scanning tunneling microscopy (ESR-STM) has allowed to acquire such information about individual spin centers of magnetic atoms bound atop a surface, while additionally providing spatial information about the binding site. Here, we conduct a full angle-dependent investigation of the hyperfine splitting for individual hydrogenated titanium atoms on MgO/Ag(001) by measurements in a vector magnetic field. We observe strong anisotropy in both the *g* factor and the hyperfine tensor. Combining the results of the hyperfine splitting with the symmetry properties of the binding site obtained from STM images and a basic point charge model allows us to predict the shape of the electronic ground state configuration of the titanium atom. Relying on experimental values only, this method paves the way for a new protocol for electronic structure analysis for spin centers on surfaces.

For decades, nuclear spins have constituted an excellent resource to gain information about the atomic scale.^1^ In recent years, advances in many different architectures, including nitrogen vacancy centers in diamond,^2^ molecular break junctions,^3^ and phosphorus donors in silicon,^4^ even allowed to address them on an individual level. This effort is mainly driven by their prospect as a future building block in quantum information processing and sensing.^5^ However, nuclear spins have been used for even longer to gain structural and electronic information about materials in bulk experiments. The nuclei can be probed directly using nuclear magnetic resonance measurements as well as indirectly via ESR because the magnitude and anisotropy of the hyperfine interaction are reflected in properties of the electron cloud surrounding the nucleus.^1^

The combination of electron spin resonance and scanning tunneling microscopy (ESR-STM) has opened a novel platform to access single nuclear spins of atoms on surfaces.^6−9^ Most strikingly, both spatial and magnetic information can be obtained by the two techniques simultaneously, providing unique access to hyperfine interaction on the atomic scale. Previous experiments showed that the hyperfine interaction of individual hydrogenated titanium (TiH) atoms on a bilayer of magnesium oxide (MgO) strongly depends on the binding side.^7^ Initial experiments hinted toward a strong anisotropic hyperfine interaction on all binding sides. However, these measurements were performed in one magnetic field direction only; this limited the electronic structure analysis and required the additional help of density functional theory (DFT) to interpret the data.^7^

Here, we perform ESR-STM measurements of individual hydrogenated Ti atoms on a bridge binding side of MgO in a vector magnetic field. We demonstrate that the hyperfine tensor has distinctly different values along its principal axes than reported previously.^7^ Combining the results from the hyperfine analysis with properties of the symmetry group of the atom’s binding site derived from STM and a basic point charge model allows us to predict the shape of the ground state orbital of the atom without the use of first-principles calculations such as DFT.

Experiments were conducted in a commercial STM system (Unisoku USM1300) equipped with a vector magnetic field (Figure 1a) and at a temperature of 1.5 K. The measurements were performed on well-isolated individual Ti atoms adsorbed on two atomic layers of MgO grown on a Ag(100) substrate. These titanium atoms were found to be hydrogenated by residual hydrogen in the vacuum chamber,^10^ effectively reducing them to Ti^3+^ with spin *S* = 1/2. Figure 1b shows a STM topography of a single hydrogenated Ti atom. For ESR experiments, a radio-frequency (RF) voltage *V*_RF_ is applied to the STM tip in addition to the DC bias voltage *V*_DC_. This RF voltage can drive transitions between the two lowest lying spin states of the Ti^3+^ atom, which is subsequently detected by changes in the tunnel current Δ*I* via magnetoresistive tunneling. For the latter, a magnetic STM tip is employed that is created by transferring several Fe atoms from the surface to the STM apex. We study hydrogenated Ti atoms adsorbed on O–O bridge sites, which come in two equivalent orientations as shown in Figure 1c: “horizontal” and “vertical”, which have an in-plane magnetic field angle with respect to the crystal lattice of 14° and 76°, respectively. This leads effectively to two different orientations of the in-plane field and thus allows for a 3-dimensional mapping of the hyperfine interaction by rotating the magnet only in a single plane (see Supporting Information Section S1).

**Figure 1:**
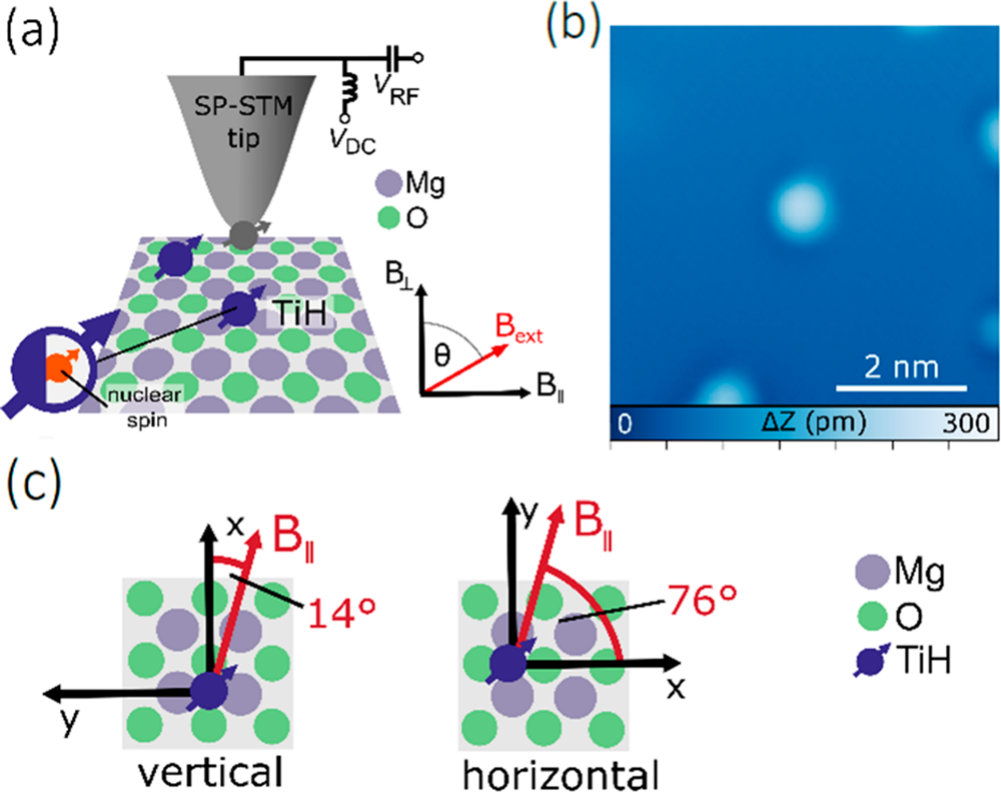
Electron spin resonance in a scanning tunneling microscope with a vector magnet. (a) Schematic of the experiment. (b) Topography image of a TiH atom on MgO (*I* = 20 pA, *V*_DC_ = 60 mV). (c) We study TiH atoms adsorbed on two equivalent bridge sites, vertical and horizontal, which effectively correspond to two different directions of the in-plane field *B*_||_.

In accordance with ref (7), we can identify three different configurations of the Ti nuclear spin. In Figure 2, we display different ESR spectra measured above atoms adsorbed on vertical bridge sites; we observe a single ESR resonance for ^46^Ti^3+^, ^48^Ti^3+^, and ^50^Ti^3+^ (*I* = 0), six resonances for ^47^Ti^3+^ (*I* = 5/2), and eight for ^49^Ti^3+^ (*I* = 7/2). In line with previous experiments, we observe a variation of the overall signal intensity for different magnetic field angles.^11^ Interestingly, for the isotopes carrying a nonzero nuclear spin, the different peaks are well-resolved when the external field is along the sample plane, with a splitting around ∼65 MHz, while they seem to merge when the field is aligned in the out-of-plane direction, with an ∼20 MHz splitting. This strong anisotropy of the hyperfine splitting is remarkable and could not be accurately determined with measurements performed along a single field direction.^7^

**Figure 2:**
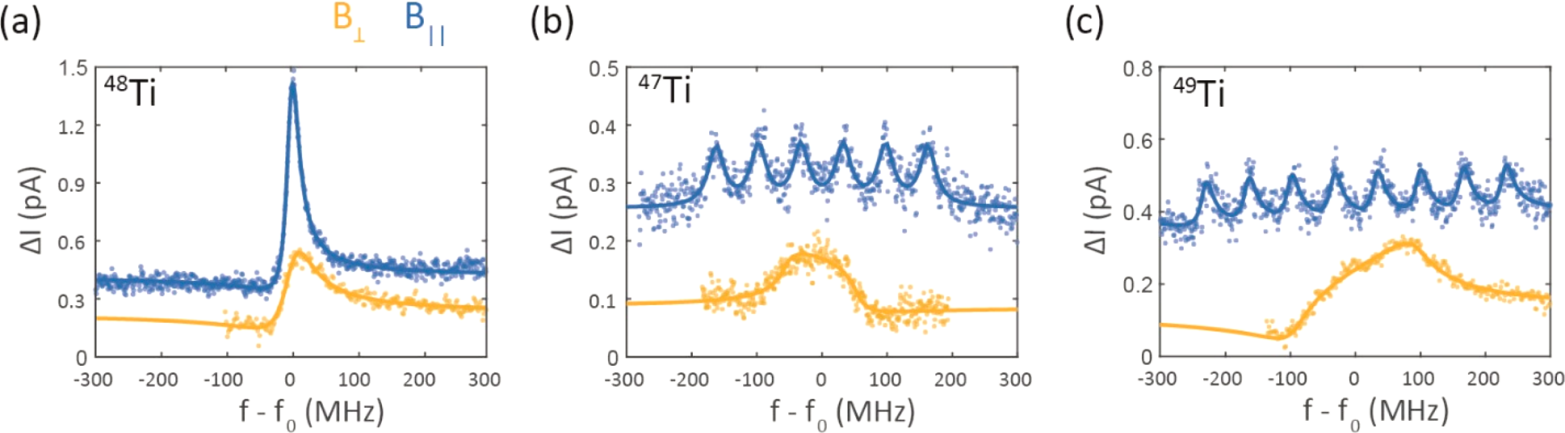
ESR spectra of different hydrogenated Ti isotopes (a, b, c) adsorbed on vertical bridge sites in an external magnetic field pointing in-plane (blue) and out-of-plane (orange). Traces were offset with respect to each other for clarity. Experimental parameters: *V*_DC_ = 60 mV, *I* = 8–10 pA, *V*_RF_ = 45–57 mV, |*B*_ext_| = 0.86–1.037 T, and *f*_0_ = 24.10–24.48 GHz.

In Figure 3, we map the full evolution of the ESR spectra as a function of θ, the angle of the magnetic field with respect to the surface normal, for two perpendicular rotation planes. Figure 3a shows data taken on a hydrogenated ^49^Ti atom on a vertical bridge site, meaning that the in-plane field makes a 14° angle with the *x*-axis. The data exhibit strong anisotropic behavior, with almost complete suppression of the hyperfine splitting for the out-of-plane field direction. All data in this panel were acquired with the same microtip, and by measuring for each data point a reference spectrum on a hydrogenated ^48^Ti atom, we can ensure that the influence of the tip field is negligible (see Supporting Information Section S1).

**Figure 3:**
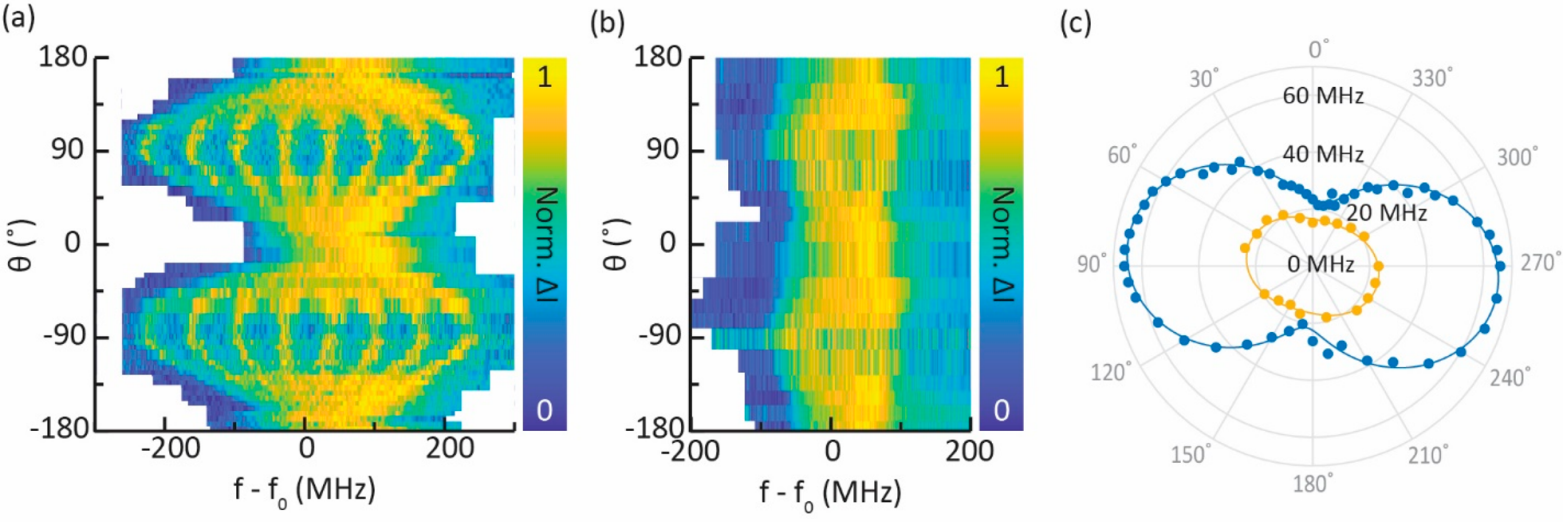
Hyperfine splitting in a vector magnetic field of hydrogenated ^49^Ti adsorbed on a vertical (a) and horizontal bridge site (b). The data in (c) are obtained by fitting each spectrum in (a) (blue dots) and (b) (yellow dots) with a sum of Fano functions (see Supporting Information Section S1), the error bars corresponding to the standard deviation of the fits are smaller than the markers’ size. Fits to the experimental data (blue and yellow lines) are based in eqs 2 and 3 (see Supporting Information Section S2).

We performed the same experiment on another hydrogenated ^49^Ti atom adsorbed on a horizontal bridge site, with a different microtip but that is again kept the same for the whole data set (see Figure 3b). Also here, we observe anisotropic behavior of the hyperfine splitting, though much less dramatic than for the vertical binding site. The evolution of the hyperfine splitting can be quantified by fitting each spectrum with several Fano functions (see Supporting Information Section S1) and is shown in Figure 3c for both adsorption sites. The evolution of the hyperfine splitting is continuous and mirror-symmetric, indicating that the sign of the magnetic field along any direction is irrelevant. We note that the observed symmetry axis is rotated by ∼10° with respect to the magnet axes. We discuss possible origins for this rotation in Supporting Information Section S2. From the anisotropic evolution of the hyperfine splitting in Figure 3c we can already infer that the extent of the ground state orbital, which scales the hyperfine splitting via the magnetic dipole–dipole interaction, is likely to be similar in two directions (out-of-plane and one in-plane) and differs substantially in the other (in-plane) one.

The anisotropy of the hyperfine splitting is closely related to that of the *g* factor. The latter had already been observed for TiH on MgO/Ag(100).^11−13^ The hyperfine interaction entails three different interactions: a dipole–dipole interaction between the electron and nuclear spins, a Fermi contact interaction that scales with the electron density at the position of the nucleus, and an orbit dipolar interaction that couples the nuclear spin and angular momentum of the unpaired electron. Spin–orbit coupling leads to a partially unquenched angular momentum which couples to the electron spin. Treating this effect up to second order with perturbation theory, one can write a spin Hamiltonian in which, in all generality, **g** and **A** are tensors:^1^
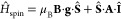
1The symmetry of the adsorption site often lowers the degree of anisotropy of these tensors for a particular set of axes (*x*, *y*, *z*). In fact, in traditional ESR spectroscopy, analysis of the hyperfine anisotropy in a vector magnetic field is used to determine the symmetry of the crystal field around the investigated species.^1,14,15^ This powerful method compensates for the lack of spatial resolution in these ensemble measurements and permits to even observe effects due to hybridization with ligand orbitals.^16^ In our case, the combination of ESR with STM allows us to measure ESR spectra of single atoms, while the symmetry of the adsorption site can be exactly determined by STM. As we show, we can thus perform an all-experimental electronic analysis to determine the shape of the ground state orbital, a quantity that has been long elusive for experimentalists.

The adsorption site of the atom has a *C*_2*v*_ symmetry (see Figure 4) so that **g** and **A** are vectors along the principal axes (*x*, *y*, *z*) of the crystal lattice.^16^ In the presence of an external magnetic field that has (*l*, *m*, *n*) directional cosines with respect to these axes, the effective *g* and *A* parameters are given by^1^
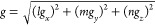
2
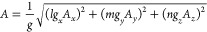
3Using these two equations, we first determine the effective *g* values for the vertical and horizontal bridge sites corresponding to different in-plane fields. We find that the vector **g** is completely anisotropic with *g*_*x*_ = 1.702 ± 0.004, *g*_*y*_ = 1.894 ± 0.004, and *g*_*z*_ = 2.011 ± 0.015. These values are in good agreement with the literature values,^11^ and the small deviations can be explained by the presence of a small residual tip field. Because this tip field has been carefully accounted for by Kim et al., we use in the following their reported *g* values.^11^ Next, we fit the data of Figure 3c to obtain the values of the hyperfine splitting, first along our field directions and, finally, along the lattice directions (see Supporting Information Section S2). We here find *A*_*x*_ = 68 ± 4 MHz, *A*_*y*_ = 18 ± 4 MHz, and *A*_*z*_ = 19 ± 4 MHz. The minima of the two data sets are each a measure of *A*_*z*_; however, they are not exactly equal. We attribute the difference, which has been taken into account for the estimation of the error in *A*_*z*_, to small variations in the local electric field surrounding the two atoms. Statistical variations of the *g* factor of Ti^3+^ atoms adsorbed on oxygen sites were indeed also observed and attributed to the same origin.^13^ The errors for the in-plane components are dominated by the uncertainty concerning the tilt of the in-plane field with respect to the crystal lattice (see Supporting Information Section S2).

**Figure 4:**
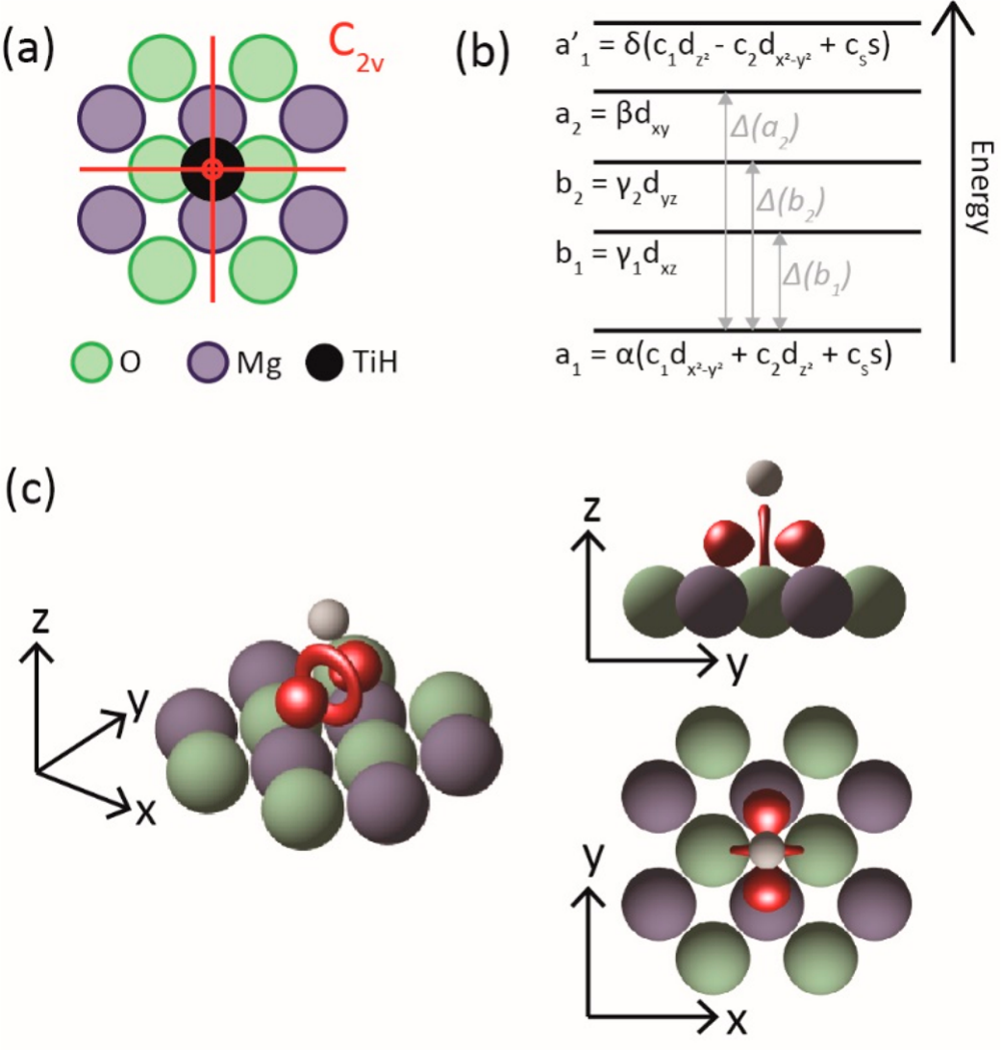
Determination of the ground state orbital. (a) Ti^3+^ is adsorbed on a bridge site with *C*_2*v*_ symmetry. (b) Energy diagram for *C*_2*v*_ symmetry.^16^ The order of the excited states is arbitrary and bears no consequence on the analysis. (c) Isosurface of the ground state orbital (red) obtained for *c*_s_ = 0. Green spheres represent O atoms, blue spheres Mg atoms, and white sphere the H atom on top of Ti.

Once both the values of **g** and **A** are determined, we can investigate how these relate to the d^1^ ground state configuration of the Ti^3+^. The corresponding energy diagram for *C*_2*v*_ symmetry is displayed in Figure 4b.^16^ The order of the excited states is arbitrarily chosen and bears no influence on the analysis. The ground state orbital is a superposition of d_*x*^2^ – *y*^2^_, d_*z*^2^_, and 4s orbitals, and our study revolves around determining the values of their respective weights *c*_1_, *c*_2_, and *c*_s_, which satisfy the normalization equation *c*_1_^2^ + *c*_2_^2^ + *c*_s_^2^ = 1. The molecular coefficients α, β, γ_1_, γ_2_, and δ quantify the hybridization of the d levels with ligand orbitals, which we assume to be small—these coefficients are therefore expected to be close to 1.

In *C*_2*v*_ symmetry, the electronic configuration of the d levels causes anisotropy of **g** in the following way:^16^
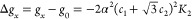
4
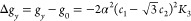
5
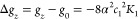
6where *g*_0_ = 2.0023, *K*_1_ = β^2^ξ/Δ(*a*_2_), *K*_2_ = γ_2_^2^ξ/Δ(*b*_2_), and *K*_3_ = γ_1_^2^ξ/Δ(*b*_1_), with ξ being the spin–orbit coupling constant and Δ(*a*_2_)[Δ(*b*_2_), Δ(*b*_1_)] the energy difference between the excited state *a*_2_ [*b*_2_, *b*_1_] and ground state *a*_1_ (see Figure 4b). As for the **A** vector we have
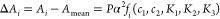
7where *i* = *x*, *y*, *z*, , *P* = *g*_0_*g*_N_μ_N_μ_B_⟨*r*^–3^⟩ (*g*_N_: nuclear *g* factor; μ_B_: electron Bohr magneton; μ_N_: nuclear Bohr magneton) scales with the radial extent of the electronic wave function via ⟨*r*^–3^⟩, and *f*_*i*_ are functions whose full expressions can be found in Supporting Information Section S3. These equations, along with the normalization condition for *c*_1_, *c*_2_, and *c*_*s*_ above, allow us to calculate the anisotropy of **g** and **A** for a given set of parameters (*P*, α, *c*_1_, *c*_2_, *c*_s_) and therefore identify all sets of parameters that could, from a symmetry argument, describe our system. We find that more than one set of parameters can lead to the experimentally observed **g** and **A** (see Supporting Information Section S3). Consequently, we employ a basic point charge model (Supporting Information Section S4) that allows us to discriminate the different solutions by their Coulomb interaction. The lateral positions of the atoms are determined experimentally by atomic resolution STM images. The positions in the *z*-direction of the Ti and H atoms are estimated, but we ensure the robustness of the model against variations of these parameters. The state with the lowest Coulomb energy is shown in Figure 4c. It consists of a superposition of the d_*x*^2^ – *y*^2^_ (74%) and d_*z*^2^_ (26%) orbitals in very good agreement with results obtained from DFT calculations.^7^ This is quite remarkable because our electronic structure analysis is solely based on experimental data assisted by the symmetry group of the surface and a basic point charge model. However, our model cannot discriminate between different values of *c*_s_ which scales the admixture of the 4s orbital (see Supporting Information Section S3). Nevertheless, we show that additional admixture of *c*_s_ merely influences the shape of the orbital by reducing the size of the central ring that points toward the neighboring O atoms (see Supporting Information Section S5).

In summary, this work illustrates how an analysis of the anisotropic hyperfine interaction can be exploited to gain an in-depth knowledge about the shape of the ground state orbital. Crucial for this method is the addition of binding site information derived from STM, which we process in a basic point charge model. Because this protocol can be applied to other spin systems on surfaces in a straightforward manner, it paves the way to determine the spin ground states of atoms and molecules on surfaces and constitutes an independent method that more elaborate theoretical methods such as DFT can be benchmarked against.

While writing this manuscript, we became aware of a similar experiment performed in another group.^17^ Overall, their results agree very well with those presented here: A strong anisotropy of the hyperfine splitting along the oxygen direction is also found in their experiment. In contrast to our work, they determine the shape of the ground state orbital via DFT, which allows to shed light onto the origin of anisotropic and isotropic contributions to the hyperfine interaction from a first-principles perspective.

## Notes

All data presented in this paper are publicly available through Zenodo.^18^

## Supporting Information Available

Details on fitting of ESR spectra, fitting of the hyperfine splitting, anisotropy of the hyperfine splitting in *C*_2*v*_ symmetry, point charge model, and influence of *c*_s_ on the ground state orbital (PDF)
