# Supplementary material for: Experimental Determination of a Single Atom Ground State Orbital through Hyperfine Anisotropy
Source: Nano Lett. 2022 Oct 28;22(21):8470–4. doi: 10.1021/acs.nanolett.2c02783 (PMC9650725; doi:10.1021/acs.nanolett.2c02783)
Supplement: Supplementary file 1 — nl2c02783_si_001.pdf [file nl2c02783_si_001.pdf]

# Supplementary Material: Experimental Determination of a Single Atom Ground State Orbital through Hyperfine Anisotropy

*Laëtitia Farinacci* †1, *Lukas M. Veldman*†1, *Philip Willke*2, *Sander Otte*1,*

1 Department of Quantum Nanoscience, Kavli Institute of Nanoscience, Delft University of Technology, 2628 CJ Delft, the Netherlands.
2 Physikalisches Institut, Karlsruhe Institute of Technology, Karlsruhe, Germany
*E-mail: corresponding author: a.f.otte@tudelft.nl
† These authors contributed equally

## Section 1. Fitting of ESR spectra

### Section 1.1. Fits with modified Fano functions

In order to limit the number of free parameters to fit the hyperfine spectra we recorded for each field direction a reference spectrum on a $^{48}$Ti atom adsorbed on the same bridge site at the same settings as those used for the $^{49}$Ti atom. The reference data is fitted with a modified Fano function [1]:

$$\Delta I = I_0 + I_1 \frac{1+\alpha\delta}{1+\delta^2} \tag{S1}$$

with $\delta = (f - f_0)/(\Gamma/2)$, where $f_0$ is the center frequency of the resonance, $\Gamma$ its width, $I_1$ its intensity, $I_0$ a current offset and $\alpha$ a parameter that accounts for the asymmetry of the resonance.

The data recorded on the hyperfine atom is then fitted with a sum of Fano functions:

$$\Delta I = \tilde{I}_0 + \sum_{i=0}^{i=n-1} \tilde{I}_1 \frac{1+\alpha\delta_i}{1+\delta_i^2} \tag{S2}$$

with $\delta_i = (f - (\tilde{f}_0 + iA))/(\Gamma/2)$, where $\tilde{f}_0$ is the center frequency of the first peak and $A$ the hyperfine splitting parameter. The parameters $\alpha$ and $\Gamma$ are fixed to the values obtained on the reference atom and $n$ is the number of resonances that is determined by the value of the nuclear spin ($n = 6$ for $I = 5/2$ and $n = 8$ for $I = 7/2$). As a result, $\tilde{f}_0$, $A$, $\tilde{I}_0$ and $\tilde{I}_1$ are the free parameters for this fit. We note that the splitting of the peaks are assumed to be identical, which means that we neglect the quadrupole interaction. This assumption is asserted when the peaks are individually resolved, i.e. for relatively large hyperfine splitting. At smaller splitting, the quadrupole interaction could compete with the hyperfine interaction [2] but this remains below our energy resolution.

## Section 1.2. Estimation of the g vector and tip field

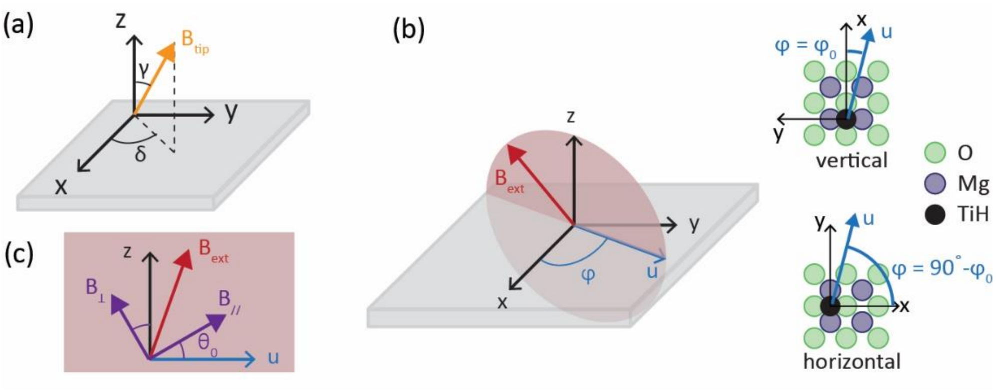


**Figure S1**. Schematics describing the angles considered in this paper. (a) The tip field is described by its amplitude, the angle $\gamma$ it makes with the $z$ axis and its azimuthal angle $\delta$ with respect to the $x$ axis. (b) The measurements span the red plane, defined by the directions of the in-plane and out-of-plane fields. The line at which it intersect the sample plane, with vector director $\vec{u}$ is at an angle $\varphi$ with the $x$ axis. More precisely, for the vertical bridge site $\varphi = \varphi_0$ and for the horizontal bridge site $\varphi = 90° - \varphi_0$. (c) In the rotation plane of the field, to reproduce our experimental observations, we furthermore consider an effective tilt angle $\theta_0$ of the magnet axes with respect to the local crystal field of the atoms.

In this section we evaluate the TiH g factor and the influence of the tip field on its evaluation. We can estimate the strength of the tip field by analyzing the position of the resonances as a function of external field. In the absence of any additional tip field the resonance position for $^{48}$Ti (and the mean position for $^{49}$Ti) is given by

$$f_0 = \frac{\mu_B}{h}\sqrt{(g_{\parallel}B_{\parallel})^2 + (g_z B_{\perp})^2} \tag{S3}$$

Where $g_{\parallel} = g_v$ for the vertical bridge site and $g_{\parallel} = g_h$ for the horizontal bridge site. Neglecting any additional field, we obtain for the vertical bridge site: $g_v = 1.688 \pm 0.005$ and $g_z = 2.005 \pm 0.006$ and, for the horizontal bridge site: $g_h = 1.906 \pm 0.009$ and $g_z = 2.017 \pm 0.009$.

Using eq. 3 of the main text these values can be compared to the literature values [1] when taking into account the tilt of our in-plane field with respect to the crystal lattice (see Figure S1) that we estimate to be $\varphi_0 = 14 \pm 2°$. This angle is given by our experimental setup and has been determined in previous experiments [3]. In Table S1 the results of our evaluation are presented, showing first a small variation within our error bars of $\varphi_0$.

| $\varphi_0$ | 12° | 14° | 16° | Kim et al. |
|---|---|---|---|---|
| $g_x$ | 1.698 | 1.702 | 1.706 | 1.653 |
| $g_y$ | 1.897 | 1.894 | 1.890 | 1.917 |
| $g_z$ | 2.011 | 2.011 | 2.011 | 1.989 |

**Table S1**. g-values obtained from our experimental data taking into account an uncertainty of 2° for $\varphi_0$ and compared to literature values [1] (right column).

We find a good agreement with the literature values and the small deviations can be explained by the presence of a small residual tip field, that has been carefully accounted for in [1]. More precisely, using from now on the g values of [1], we consider a field that has a fixed magnetization direction and can switch along its axis so that $\boldsymbol{B}_{tip} \cdot \boldsymbol{B}_{ext} > 0$ [1]. We describe the tip field by its amplitude $|\boldsymbol{B_{tip}}|$ and the angle it makes with respect to the surface normal, $\gamma$, as well as its azimuthal angle with the x axis, $\delta$ (see Figure S1a).

We find for the microtip used for the vertical bridge site data (the error bars correspond to the 95% confidence interval of the fit coefficients)

| $\varphi_0$ | 12° | 14° | 16° |
|---|---|---|---|
| $\lvert\boldsymbol{B}_{tip}\rvert$ | $100 \pm 30$ mT | $110 \pm 40$ mT | $80 \pm 40$ mT |
| $\gamma$ | $101 \pm 4°$ | $111 \pm 4°$ | $106 \pm 7°$ |
| $\delta$ | $91 \pm 3°$ | $89 \pm 1°$ | $90 \pm 2°$ |

**Table S2**. Determination of the tip-field for the micro-tip used to record the data taken on Ti atoms adsorbed on a vertical bridge site; whilst considering our error bars of $\varphi_0$.

And for the horizontal data set we have (we used $\delta = -\varphi_0$ since the value obtained for $g_h$ corresponds to the effective value obtained from Ref. [1] along the field direction)

| $\varphi_0$ | 12° | 14° | 16° |
|---|---|---|---|
| $\lvert\boldsymbol{B}_{tip}\rvert$ | $11 \pm 5$ mT | $12 \pm 5$ mT | $13 \pm 5$ mT |
| $\gamma$ | $25 \pm 155°$ | $15 \pm 105°$ | $5 \pm 60°$ |

**Table S3**. Determination of the tip-field for the micro-tip used to record the data taken on Ti atoms adsorbed on a horizontal bridge site; whilst considering our error bars of $\varphi_0$.

## Section 2. Fitting of the hyperfine splitting

### Section 2.1. Determination of the hyperfine values

Based on eq. (3) of the main text we fit the data of Fig.3c of the main text with the following function

$$A = \frac{1}{g}\sqrt{\tilde{l}^2 g_{v,h}^2 A_{v,h}^2 + n^2 g_z^2 A_z^2} \qquad \text{(S4)}$$

Where $\tilde{l}$ and $n$ are the cosine directions of the external field along the $u$ and $z$ axis respectively (see Figure S1b). As mentioned in the main text, we observe a rotation of the data in Fig.3c with respect to the magnet axes. Possible origins for this effective tilt are discussed in the next subsection. We account for it by an effective tilt between the magnet axes and the crystal field axes of the atom. More precisely, we consider an offset angle $\theta_0$ between $(\boldsymbol{B}_{\parallel}, \boldsymbol{B}_{\perp})$ and $(\boldsymbol{u}, \boldsymbol{z})$ (see Figure S1c) and we therefore have

$$\tilde{l} = \cos(\theta - \theta_0) \qquad \text{(S5)}$$

$$n = \sin(\theta - \theta_0) \qquad \text{(S6)}$$

where $\tan\theta = \frac{B_\perp}{B_\parallel}$.

The fits in Fig.3c of the main text are based on eq.(S4)-(S6) and show a very good agreement with the experimental data. We obtain for the vertical bridge site $\theta_0 = -6.8° \pm 0.8°$, $A_v = 65.4 \pm 0.7$ MHz, $A_z = 21.7 \pm 1$ MHz and for the horizontal bridge site $\theta_0 = -15° \pm 5°$, $A_h = 23.6 \pm 0.8$ MHz, $A_z = 16.1 \pm 0.8$ MHz.

Taking into account the presence of a tip-field, as determined in the previous section, does not improve the quality of the fits and leads to variations of less than 1 MHz of the fit coefficients. In particular, the presence of a tip field cannot account for the different values of $A_z$. These are most likely due to local variations of the electric field for each atom as also observed in Ref. [4].

To obtain the values of the hyperfine vector along the lattice direction we again have to take into account the tilt of the in-plane field with respect to the crystal lattice. Using eq. (3) of the main text we have

| $\varphi_0$ | 12° | 14° | 16° |
|---|---|---|---|
| $A_x$ | $67 \pm 2$ MHz | $68 \pm 2$ MHz | $69 \pm 2$ MHz |
| $A_y$ | $20.5 \pm 1.5$ MHz | $19 \pm 2$ MHz | $17 \pm 2$ MHz |

**Table S4**. Values for hyperfine splitting along the x and y axis when taking into account the error bars for $\varphi_0$.

As one can see, the uncertainty concerning $\varphi_0$ dominates the error bars for $A_x$ and $A_y$.

### Section 2.2. Possible origins of the rotation

As discussed in the main text and the last section, we observe an offset $\theta_0 \sim 10°$ between the symmetry axis of the measured data and the magnet axes. In this section, we consider several origins for this observation: tip magnetic field, tilt of the STM head, tilt of the sample, local variations in the electric field emerging from inhomogeneities of the substrate, and electrostatic forces emanating from the tip.

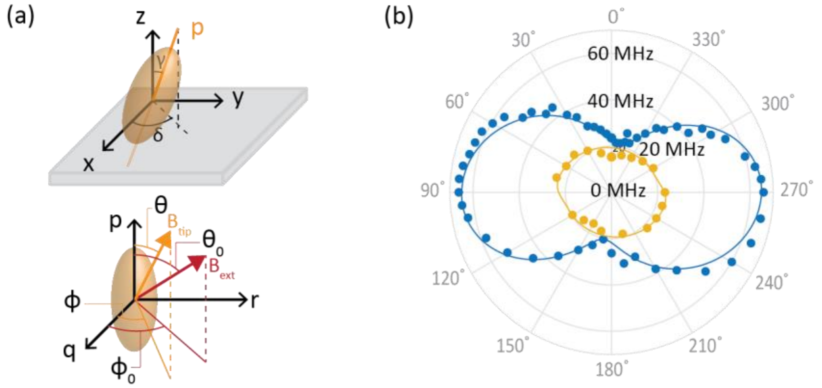


**Figure S2**. (a) Description of the tip field by the Stoner-Wohlfarth model. The orientation of its easy axis *p* is defined by the angle $\gamma$ that makes with the *z* axis, and its azimuthal angle $\delta$ with the *x* axis (top). Around the easy axis, the tip and magnetic fields make an angle $\theta$ and $\theta_0$ with *p* and have azimuthal angles $\phi$ and $\phi_0$ (bottom). The orientation of $\boldsymbol{B_{tip}}$ is then determined by minimizing the energy of the magnet. (b)

Fitting of the experimental data to account for the rotation with respect to the magnet axes using a tip magnetic field described by the Stoner-Wohlfarth model (note that contrary to Fig.3c of the main text, the values of $A_x$, $A_y$ and $A_z$ are not fit parameters but fixed).

In order to investigate if the tip magnetic field could be at the origin of the observed rotation between the data of Fig.3c and the magnet axes we modelled the tip field with a Stoner-Wohlfarth model. This model allows for the tip magnetization to deviate from its main axis and therefore covers a wider range of magnet behaviors in an external field than the model considered in the previous section. More precisely, the orientation of the easy axis of the magnet, *p*, with respect to the (x,y,z) basis is characterized by its angle $\gamma$ with the z axis, and its azimuthal angle $\delta$ with respect to the *x* axis (see Fig.S2a). The behavior of the magnet is assumed to be isotropic around this axis. Considering a basis (p,q,r) around this axis the orientation of the magnet and external fields are characterized by their angles with respect to the *p* axis, $\theta$ and $\theta_0$, and their azimuthal angles, $\phi$ and $\phi_0$, respectively. The energy E of the system is then given by

$$E = \frac{V\,B_{tip}}{2}[-H_k \cos^2\theta - 2B_{ext}(\sin\theta_0 \sin\theta \cos\phi_0 \cos\phi + \sin\theta_0 \sin\theta \sin\phi_0 \sin\phi + \cos\theta_0 \cos\theta)] \quad \text{(S7)}$$

where V is the volume of the magnet and $H_k$ is the anisotropy field. The first term is the magnetic anisotropy and the second the Zeeman energy. The orientation of the tip field is then obtained by determining the minima of E, i.e. by solving $\frac{\partial E}{\partial \phi} = 0$ and $\frac{\partial E}{\partial \theta} = 0$. When two minima are found we weight each solution by their Boltzmann population at 1K.

Using this model, we can estimate which tip field would be needed to reproduce the rotation observed between the experimental data and the magnet axes. More precisely, we fit the experimental data using eq.(2) and (3) of the main text, where we fix $(g_x, g_y, g_z) = (1.653, 1.917, 1.989)$, and $(A_x, A_y, A_z) = (68, 18, 19)$. The total magnetic field is the sum of the external field (with the out-of-plane and in-plane components aligned with the *z* and *u* axes) and tip field - the latter being determined via the Stoner-Wohlfarth model. We estimate that the volume of the magnet is that of a sphere with a radius of 2 Å and set $H_k = 100$ T (we find empirically that this value of $H_k$ gives better fits to our experimental data). The fits for both adsorption sites are shown in Fig.S2b and yield, for the vertical bridge site, $B_{tip} = 0.565 \pm 0.125$ T, $\gamma = 82 \pm 3°$ and $\delta = -67 \pm 2°$; and for the horizontal bridge site, $B_{tip} = 0.270 \pm 0.670$ T, $\gamma = 13 \pm 22°$ and $\delta = -6 \pm 106°$. The fits demonstrate that a tip field can induce an apparent rotation of the data with respect to the magnet axes but do not completely reproduce the shape of the experimental data. Additionally, the tip fields corresponding to the fits would shift the ESR resonance frequencies $f_0$ substantially and therefore are too high to be realistic – by a factor $\sim 5$ for the vertical bridge site and $\sim 20$ for the horizontal bridge site (see section S1.2).

We identify a number of other possible origins for the tilt in the measurements for Fig 3c. We estimate the uncertainty of the STM head tilt with respect to the external field to be <5° based on the geometry of the STM design. The misalignment of the sample with respect to the scanning piezo was measured to be ~-0.2° in each direction. We therefore expect that although these errors could accumulate, macroscopic origins alone should be insufficient to explain the magnitude of the observed rotation.

We also consider possible origins of a microscopic nature. Local variations in the electric field emerging from inhomogeneities of the substrate have been linked to variations in the g-factor of TiH atoms

adsorbed on O-sites of the MgO lattice. The g-factor was found to be especially susceptible to changes in the in-plane direction of the electrostatic field leading to variations up to 15% [4]. This indicates that local charges play a large role in the crystalline environment the atom experiences.

Next to that, due to the anisotropic shape of the tip, the in-plane components of van der Waals and electrostatic forces emanating from the tip could move the atom slightly with respect to the crystal lattice. Similar in-plane tilting effects have been observed for molecules adsorbed on AFM tips [5] and in mechanical bond breaking experiments [6], and has been used for lateral atomic manipulation [7,8]. The driving mechanism behind ESR-STM itself has been attributed to the electric field inducing movement of the adatom via a piezo crystalline effect in the MgO [9,10].

While we cannot definitively identify the origin of the observed rotation, we believe that the effects discussed above may each contribute to its explanation. Independent of the origin, the hyperfine values and their error bars determined by the minima and maxima of the hyperfine splitting in Figure 3c remain unaffected.

## Section 3. Anisotropy of the hyperfine splitting in $C_{2v}$ symmetry

The anisotropy of the g and A vectors in $C_{2v}$ symmetry is given by eq. (7) of the main text where the functions $f_x$, $f_y$ and $f_z$ are defined as follows [11 p.382]

$$f_x = -\frac{4}{3}\left(c_1+\sqrt{3}c_2\right)^2 K_2 + \frac{2}{3}\left[\left(c_1-\sqrt{3}c_2\right)^2 K_3 + 4c_1^2 K_1\right] + \frac{2}{7}\left(c_1^2-c_2^2-2\sqrt{3}c_1c_2\right) - \frac{4\sqrt{3}}{7}c_1c_2K_1 + \frac{\sqrt{3}}{7}\left(\sqrt{3}c_1+c_2\right)\left(c_1-\sqrt{3}c_2\right)K_3 \quad \text{(S8)}$$

$$f_y = -\frac{4}{3}\left(c_1-\sqrt{3}c_2\right)^2 K_3 + \frac{2}{3}\left[\left(c_1+\sqrt{3}c_2\right)^2 K_2 + 4c_1^2 K_1\right] + \frac{2}{7}\left(c_1^2-c_2^2+2\sqrt{3}c_1c_2\right) + \frac{4\sqrt{3}}{7}c_1c_2K_1 + \frac{\sqrt{3}}{7}\left(\sqrt{3}c_1-c_2\right)\left(c_1+\sqrt{3}c_2\right)K_2 \quad \text{(S9)}$$

$$f_z = -\frac{16}{3}c_1^2K_1 + \frac{2}{3}\left[\left(c_1+\sqrt{3}\,c_2\right)^2 K_2 + \left(c_1-\sqrt{3}\,c_2\right)^2 K_3\right] - \frac{4}{7}\left(c_1^2-c_2^2\right) - \frac{\sqrt{3}}{7}\left(\sqrt{3}\,c_1+c_2\right)\left(c_1-\sqrt{3}\,c_2\right)K_3 - \frac{\sqrt{3}}{7}\left(\sqrt{3}\,c_1-c_2\right)\left(c_1+\sqrt{3}\,c_2\right)K_2 \quad \text{(S10)}$$

We calculate the values of $\Delta A_x$, $\Delta A_y$ and $\Delta A_z$ for parameter sets $(P, \alpha, c_1, c_2, c_s)$ where P spans $[0; -200]$ MHz, $\alpha$ spans [0;1], $c_s$ spans $[0; 0.8]$ for which we only calculate sets in increments of 0.2. $c_1$ and $c_2$ are calculated via the normalization equation $c_1^2 + c_2^2 + c_s^2 = 1$. An angle $\chi$ is defined as $\tan\chi = \frac{c_2}{c_1}$, where $\chi$ spans $[0°; 360°]$. The calculation is performed in the following way: first the values of $K_1$, $K_2$, and $K_3$ are calculated via eq. (4)-(6) of the main text and then $\Delta A_x$, $\Delta A_y$ and $\Delta A_z$ are obtained from eq. (7), (S8)-(S10).

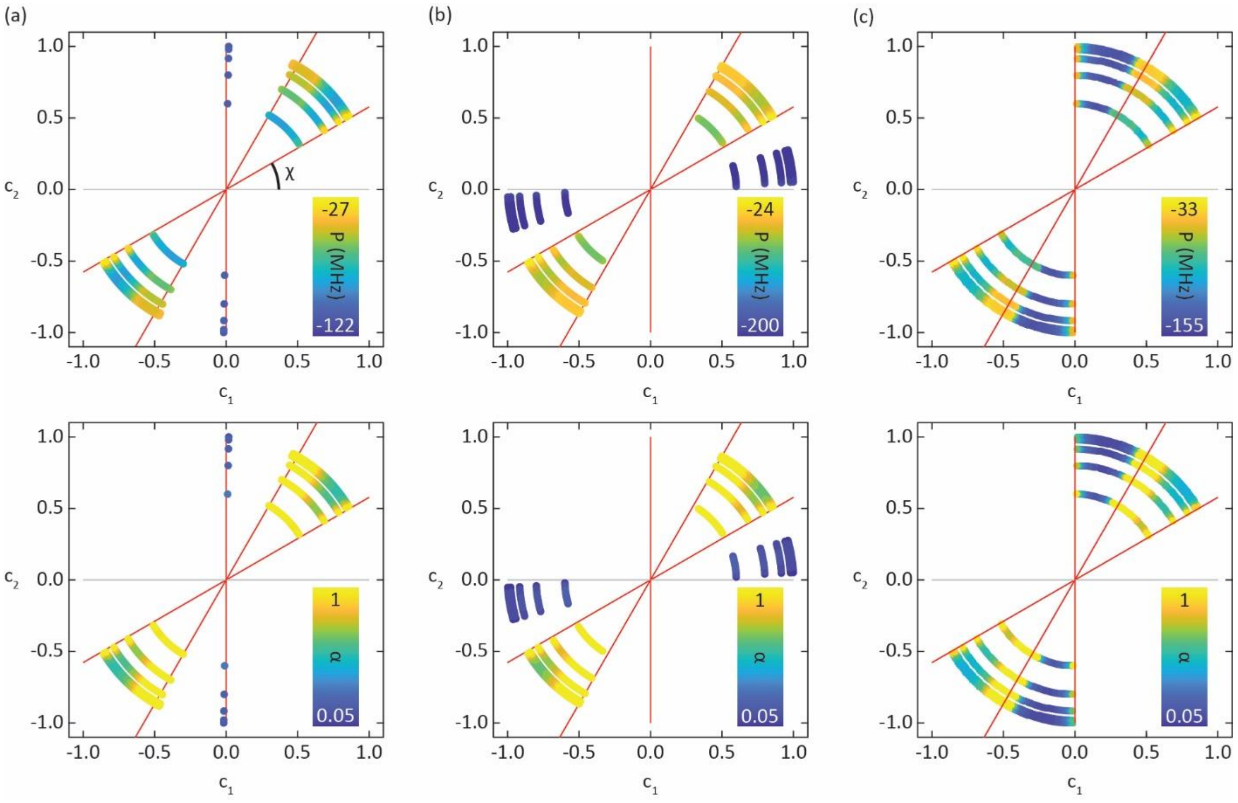


**Figure S3**. Sets of parameters $(P, \alpha, c_1, c_2, c_s)$ that can give rise the observed anisotropy of the hyperfine splitting. (a-c) considers additionally variations of **g** (a) Values as reported in Ref. [1]: $(g_x, g_y, g_z) = (1.653, 1.917, 1.989)$. (b) $(g_x, g_y, g_z) = (1.655, 1.898, 2.013)$. (c) $(g_x, g_y, g_z) = (1.651,\ 1.936,\ 1.965)$.

A parameter set is considered to be a valid solution if the values obtained for $\Delta A_x$, $\Delta A_y$ and $\Delta A_z$ are within $\pm 4$ MHz of the experimental values. We plot in Fig. S3a the data points that correspond to such valid solutions: each solution is represented with a point whose coordinates are $(c_1, c_2)$ and the value of $c_s$ determines the distance between this point and the origin (because of the normalization equation) - as a result the different concentric cycles correspond to the different values of the $c_s$ parameter. The two remaining parameters, $P$ and $\alpha$, are represented by the color of the points in upper and lower graphs, respectively. We ensured the stability of our model against the uncertainty of **g** reported in literature and Fig. S3b-c, shows two examples of the results of the calculations performed when considering a combination of extrema/minima of the components of **g** (we performed the calculation for all 8 possible combinations).

As one can see, the value of $c_s$ does not discriminate between different $\chi$ values but rather renormalizes the values of $\alpha$ and $P$. The two lines corresponding to $\chi = 30°$ and $\chi = 60°$ indicated by red lines are robust against variations of **g** and correspond to reasonable values for $\alpha$ and $P$. Indeed, $\alpha$ quantifies the hybridization of the d levels with ligands orbitals and, since, we assume this effect to be minor, $\alpha$ should be close to 1. On the other hand, $P$ scales with $\langle r^{-3} \rangle$ and large values of $P$ correspond to orbitals with a very small spatial extent, from literature values [11 – Table 9.13 p.359] we expect $P \sim -78$ MHz for a $Ti^{3+}$.

## Section 4. Point charge model

| Ion | Δ electrons (no.) | X(Å) | Y(Å) | Z (Å) |
|---|---|---|---|---|
| H | 1 | 0 | 0 | 1.8 |
| O | 2 | -1.45 | 0 | -1.6 |
| O | 2 | 1.45 | 0 | -1.6 |
| Mg | -2 | 0 | -1.45 | -1.6 |
| Mg | -2 | 0 | 1.45 | -1.6 |

**Table S5**. Point charge model used to identify the ground state orbital. Shown are the local charges as well as their position in the $(x, y, z)$ coordinate system centered around the Ti atom (see Figure 1 of the main text).

To discriminate between the different solutions shown in Fig. S3, we use additionally a point charge model defined from Table S5. The point charge model allows to distinguish solutions that yield the correct hyperfine values (Fig. S3), but are unlikely ground states, since the orbital charges are pointing in unfavorable directions of the surrounding crystal field. Each charge $q_i$ at a position $(x_i, y_i, z_i)$ yields a potential

$$V_i = \frac{q_i}{\sqrt{(x-x_i)^2+(y-y_i)^2+(z-z_i)^2}} \tag{S11}$$

So that the total Coulomb energy for an electron in an orbital $|\psi\rangle$ is $E_C = -e\langle\psi|\sum_i V_i|\psi\rangle$.

For each set of parameters that yields correct values for the anisotropy of the hyperfine vector we calculate the corresponding ground state orbital

$$|\psi\rangle = c_1\, d_{x^2-y^2} + c_2 d_{z^2} + c_s 4s \tag{S12}$$

where $d_{x^2-y^2}$, $d_{z^2}$ and 4s are the spherical harmonics for which the radial parts verify

$$R_{3,2} = \frac{4}{81\sqrt{6}}\left(\frac{Z_{3d}}{a_0}\right)^{3/2} \rho^2 \exp\left(-\frac{\rho}{3}\right) \tag{S13}$$

$$R_{4,0} = \frac{1}{96} Z_{4s}^{3/2}\left[24 - \frac{26\rho}{2} + 12\left(\frac{\rho}{2}\right)^2 - \left(\frac{\rho}{2}\right)^3\right] \exp\left(-\frac{\rho}{4}\right) \tag{S14}$$

where $\rho = Zr/a_0$, with $a_0$ being the Bohr radius and $Z_{3d}$ ($Z_{4s}$) the effective nuclear charge for the $3d$ ($4s$) shell.

And we have for the angular parts

$$Y_{x^2-y^2} = \frac{\sqrt{15}}{4\sqrt{\pi}} \frac{x^2-y^2}{r^2} \tag{S15}$$

$$Y_{z^2} = \frac{\sqrt{5}}{4\sqrt{\pi}} \frac{3z^2-r^2}{r^2} \tag{S16}$$

$$Y_{4s} = \frac{1}{\sqrt{4\pi}} \tag{S17}$$

Furthermore, we have (see main text)

$$P = g_0 \, g_N \, \mu_N \, \mu_B \, \langle r^{-3} \rangle \tag{S18}$$

Neglecting for simplicity any contribution of $c_s$, the radial extent of the orbital can be calculated from the radial wave-function for $d$ orbitals [see eq. S(13)]

$$\langle r^{-3} \rangle = \int_0^\infty \left[R_{3,2}(r)\right]^2 r^{-3} r^2 dr \tag{S19}$$

And we obtain

$$\langle r^{-3} \rangle = \frac{Z^3}{81 a_0^3} \tag{S20}$$

Therefore, for each set of solutions determined after the previous step, we calculate the value of $Z_{3d}$ using the value of the $P$ parameter and eq. (S18) and (S20). The effective nuclear charge for the $4s$ orbital is then adjusted so that the ratio $\frac{Z_{3d}}{Z_{4s}}$ equals the one given in literature [12].

The calculation is performed using a grid in the $(x, y, z)$ space that spans $[-4a_0: 4a_0]$ in each direction and with a spacing of $0.1a_0$ between points. We ensure robustness of the results by varying the position of the Ti atom with respect to the crystal lattice along the z direction in the range of 20%. The position of the Mg and O atoms are determined experimentally by atomic resolution images and the one of the H atom is set according to [13].

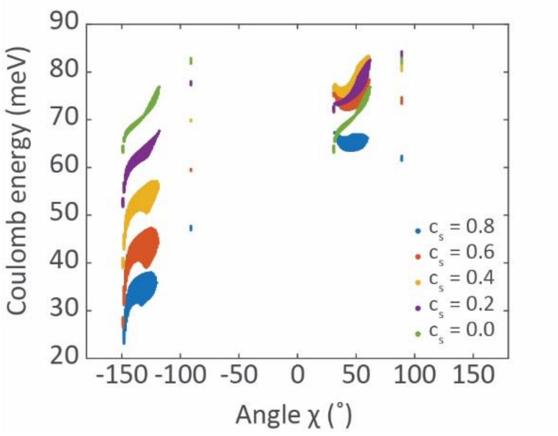


**Figure S4**. Coulomb energy for the different sets of parameters that correctly describe the hyperfine anisotropy.

In Figure S4 we show the Coulomb energy calculated for the sets of parameters shown in Figure S4a. Each color represents a different value of $c_s$ and the multiplicity of points for given $\chi$ and $c_s$ values corresponds to the multiple sets of candidates that contains these values. As one can see, decreasing $c_s$ leads to a systematic decrease in the Coulomb energy. While this can be easily explained by the smaller radial extent of the $4s$ orbital with respect to the $3d$ orbitals, the calculation suggests that the minimal solution corresponds to an electron only localized in the $4s$ orbital which is unrealistic. The point charge model therefore does not allow to determine with certainty the value of $c_s$. However, it allows us to clearly identify the ground state orbital for each value of $c_s$. As we consistently find a minimum at $\chi = -150°$

for every value of $c_s$ we conclude that the relative mixture between the *d* orbitals is not affected by the addition of $c_s$.

## Section 5. Influence of $c_s$ on the ground state orbital

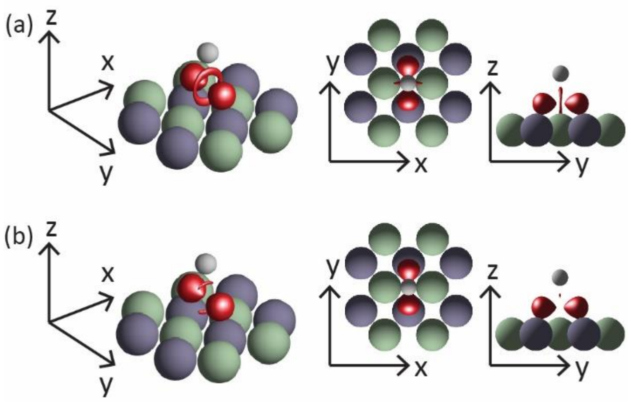


**Figure S5**. Influence of $c_s$ on the ground state orbital. Solution obtained for $c_s = 0.2$ (a) and $c_s = 0.4$ (b).

In the main text, we show the solution obtained for $c_s = 0$. In Figure S5, we show the optimal solutions for $c_s = 0.2$ (a), which is most likely an upper boundary for the admixture of the $4s$ orbital, as well as for $c_s = 0.4$ (b), which is unrealistic but help us better capture the influence of the parameter. The admixture of the $4s$ orbital influences only marginally the shape of the orbital: it mostly reduces the size of the central ring that points towards the neighboring O atoms. We ensured that these results are robust against variations of the g vector within the error bars given in Ref. [1].

REFERENCES


1. Kim, J.; Jang, W. J.; Bui, T. H.; Choi, D. J.; Wolf, C.; Delgado, F.; ... & Bae, Y. (2021). Spin resonance amplitude and frequency of a single atom on a surface in a vector magnetic field. *Physical Review B* **2021**, *104* (17), 174408. Link

2. Willke, P.; Bae, Y.; Yang, K.; Lado, J. L.; Ferrón, A.; Choi, T.; Ardavan, A.; Fernández-Rossier, J.; Heinrich, A.J.; Lutz, C.P. Hyperfine interaction of individual atoms on a surface. *Science* **2018**, *362* (6412), 336-339. Link

3. Veldman, L.; Farinacci, L.; Rejali, R.; Broekhoven, R.; Gobeil, J.; Coffey, D.; Ternes, M.; Otte, A.F. Free coherent evolution of a coupled atomic spin system initialized by electron scattering. *Science* **2021**, *372* (6545), 964-968. Link

4. Steinbrecher, M.; Van Weerdenburg, W. M.; Walraven, E. F.; Van Mullekom, N. P.; Gerritsen, J. W.; Natterer, F. D., ... & Khajetoorians, A. A. Quantifying the interplay between fine structure and geometry of an individual molecule on a surface. *Physical Review B* **2021**, *103* (15), 155405. Link

5. Néel, N.; Kröger, J. Atomic force extrema induced by the bending of a CO-functionalized probe. *Nano Letters* **2021**, 21(5). Link

6. Chen, P.; Fan, D.; Zhang, Y.; Selloni, A.; Carter, E.A.; Arnold, C.B.; Dankworth, D.C.; Rucker, S.P.; Chelikowsky, J.R.; Yao, N. Breaking a dative bond with mechanical forces. *Nature communications*. **2021**, 12(1). Link

7. Stroscio, J. A.; Celotta, R. J. Controlling the dynamics of a single atom in lateral atom manipulation. *Science* **2004**, 306(5694). Link

8. Ternes, M.; Lutz, C.P.; Hirjibehedin, C.F.; Giessibl, F.J.; Heinrich, A.J. The force needed to move an atom on a surface. *Science* **2008**, 319(5866). Link

9. Lado, J.L.; Ferrón, A.; Fernández-Rossier, J. Exchange mechanism for electron paramagnetic resonance of individual adatoms. *Physical Review B,* **2017**, 96(20). Link

10. Seifert, T.S.; Kovarik, S.; Juraschek, D.M.; Spaldin, N.A.; Gambardella, P.; Stepanow, S. Longitudinal and transverse electron paramagnetic resonance in a scanning tunneling microscope. *Science advances* **2020,** 6(40). Link

11. Mabbs, F. E.; Collison, D. *Electron paramagnetic resonance of d transition metal compounds* (Vol. 16). Elsevier: Amsterdam, 2013.

12. Clementi, E.; Raimondi, D. L.. Atomic Screening Constants from SCF Functions. *J. Chem. Phys.* **1963**, *38* (11), 2686. Link

13. Launila, O.; Lindgren, B. Spectroscopy of TiH: Rotational analysis of the $^4\Gamma \rightarrow X^4\Phi(0,0)$ band at 530 nm. *J. Chem. Phys.* **1996**, 104(17). Link
